# Case Report: Spontaneous Fetal Demises at Third Trimester of Pregnancy Due to a Double Lopped Nuchal Cord in *Camelus dromedarius*

**DOI:** 10.3389/fvets.2022.872383

**Published:** 2022-05-24

**Authors:** Young-Bum Son, Mohammad Shamim Hossein, Xianfeng Yu, Yeon Ik Jeong, P. Olof Olsson, Woo Suk Hwang

**Affiliations:** ^1^UAE Biotech Research Center, Abu Dhabi, United Arab Emirates; ^2^Jilin Provincial Key Laboratory of Animal Model College of Animal Science, Jilin University, Changchun, China; ^3^Department of Biology, North-Eastern Federal University, Yakutsk, Russia

**Keywords:** nuchal cord, abortion, third trimester, clone, camels

## Abstract

The umbilical cord acts as the critical lifeline of the developing fetus by providing nutrients and oxygen to it. Umbilical cord abnormalities are considered the leading cause of stillbirth in humans, but information on stillbirths associated with umbilical cord abnormalities is very scant in the clinical practice of animals. Here, we described a case of fetal demise in camels indicated to be caused by fetal death from strangulation by its umbilical cord, which is commonly known as the nuchal cord. A pregnant camel at its 36 weeks of gestation spontaneously aborted a single fetus. The camel was 5 years old and nullipara. A 6-day-old cloned embryo was transferred transcervically to the recipient. Pregnancy was confirmed 50 days after embryo transfer by ultrasonography, and the pregnant camel was maintained under a standard nutritional plan. The neck of the aborted fetus was strangulated tightly by a double loop of the umbilical cord. There was no congenital anomaly or other malformation in the fetus. We concluded that the nuchal cord was tightly coiled around the neck of the fetus and interfered with the blood flow in the fetus by collapsing the umbilical vein and subsequently causing fetal death and abortion. To the authors' knowledge, this is the first reported case of a nuchal cord in camels.

## Introduction

Intrauterine life is maintained by the umbilical cord, which is the conduit between the developing fetus and the placenta. The fetus receives oxygenated and nutrient-rich blood from the placenta through umbilical veins, and umbilical arteries send back low oxygen and nutrient-depleted blood to the placenta. Any kind of lesions to the umbilical cord originating from structural, mechanical, hamartomatous, infectious, or other damage may lead to an indisputable cord failure which eventually prejudices the life or wellbeing of the fetus (1).

A nuchal cord develops when the umbilical cord becomes wrapped around the fetal neck. The prevalence of nuchal cord is very common in humans, and at parturition, it ranges between 6 and 36% (2, 3). It could have single or multiple loops and be loose or tightly wrapped. Shui and Eastman (4) studied 1,007 human infants at delivery and reported that 20.6% of the infants had a single loop, 2.5% had a double loop, and 0.2% had a triple loop. The impact of nuchal cord on the wellbeing of infants depends on the number and tightness of loops. A loosely wrapped or single-looped nuchal cord usually has a freely sliding pattern and is not associated with perinatal morbidity and mortality (2, 5), while a tightly wrapped nuchal cord has an effect similar to strangulation (1).

The predisposing cause of the nuchal cord could be the uterine, fetal, and/or umbilical cord biometrics, such as length of the umbilical cord, volume of placental and amniotic fluid, and fetal movement in the uterus (1). Sherer et al. (6) reported that excessively long umbilical cords, polyhydramnios, and excessive fetal movement may be the leading cause of the nuchal cord. Nuchal cord in humans can be diagnosed by ultrasonic examination, and necessary measures could be taken according to the type of nuchal cord and length of gestation. In farm animals, there is no report of nuchal cord of any type. This report describes the abortion of a fetus derived by somatic cell nuclear transfer due to strangulation caused by a double-looped nuchal cord.

## Case Presentation

**Figure 1:**
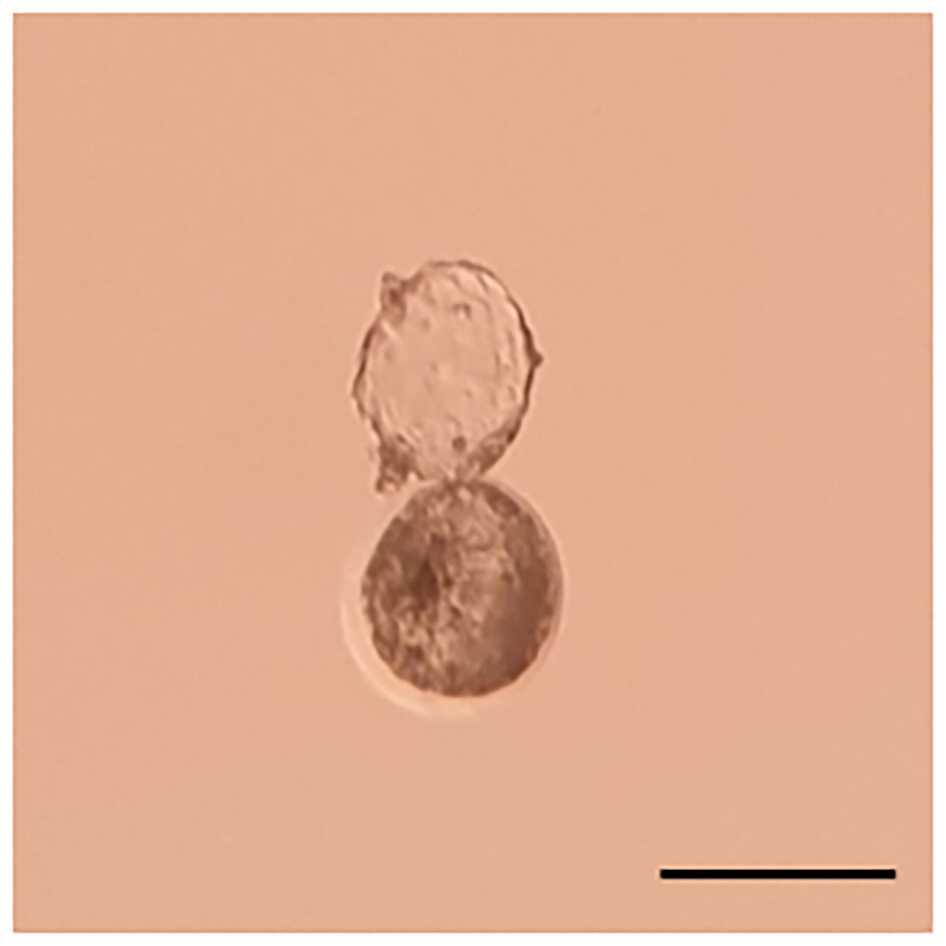
Photograph of the blastocyst transferred to the recipient. The blastocyst was 6 days old and started to hatch through the zona pellocida. The bar is 250 um.

**Figure 2:**
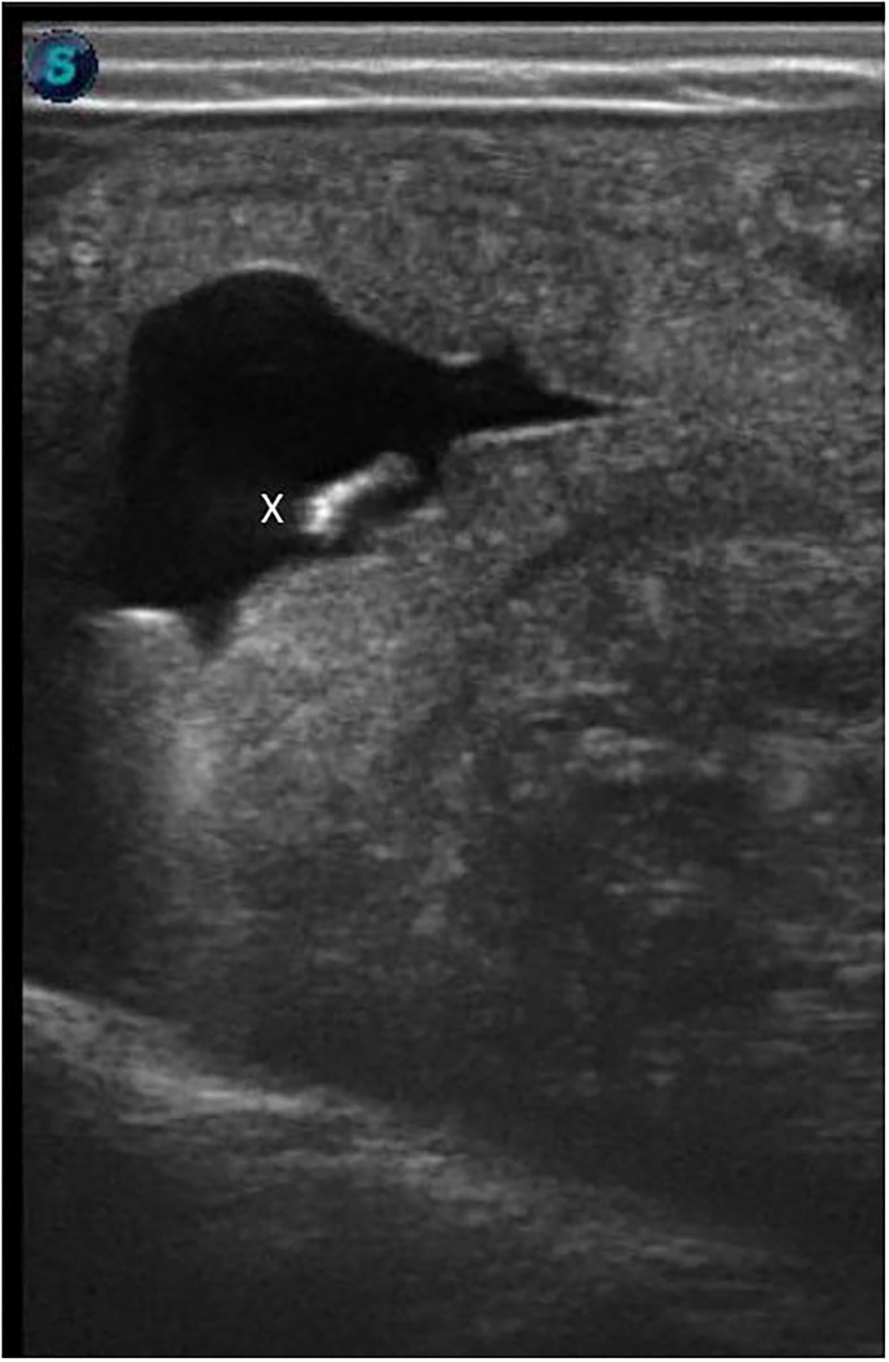
Ultrasonographic confirmation of pregnancy after transfer of cloned blastocyst. The ultrasonographic image shows a 56-day-old fetus (x) on the uterine wall.

A 36-week pregnant camel (chip number: 123654896571233, Nullipara, body weight 450 kg) spontaneously aborted a single fetus in a large-scale cloning facility. The camel received a single 6 day old embryo produced by somatic cell nuclear transfer using *in vivo* matured oocytes. A photograph of the transferred embryo is presented in Figure 1. We have followed our standard laboratory procedure for collection of tissue from somatic cell donors, the establishment of fibroblast cell line, super stimulation of oocyte donor, synchronization of oocyte donor and recipient camels, embryo transfer, and pregnancy diagnosis (7–9). Pregnancy was confirmed 50 days after embryo transfer by ultrasonography (Figure 2). The camel was maintained under a standard nutrition plan.

**Figure 3:**
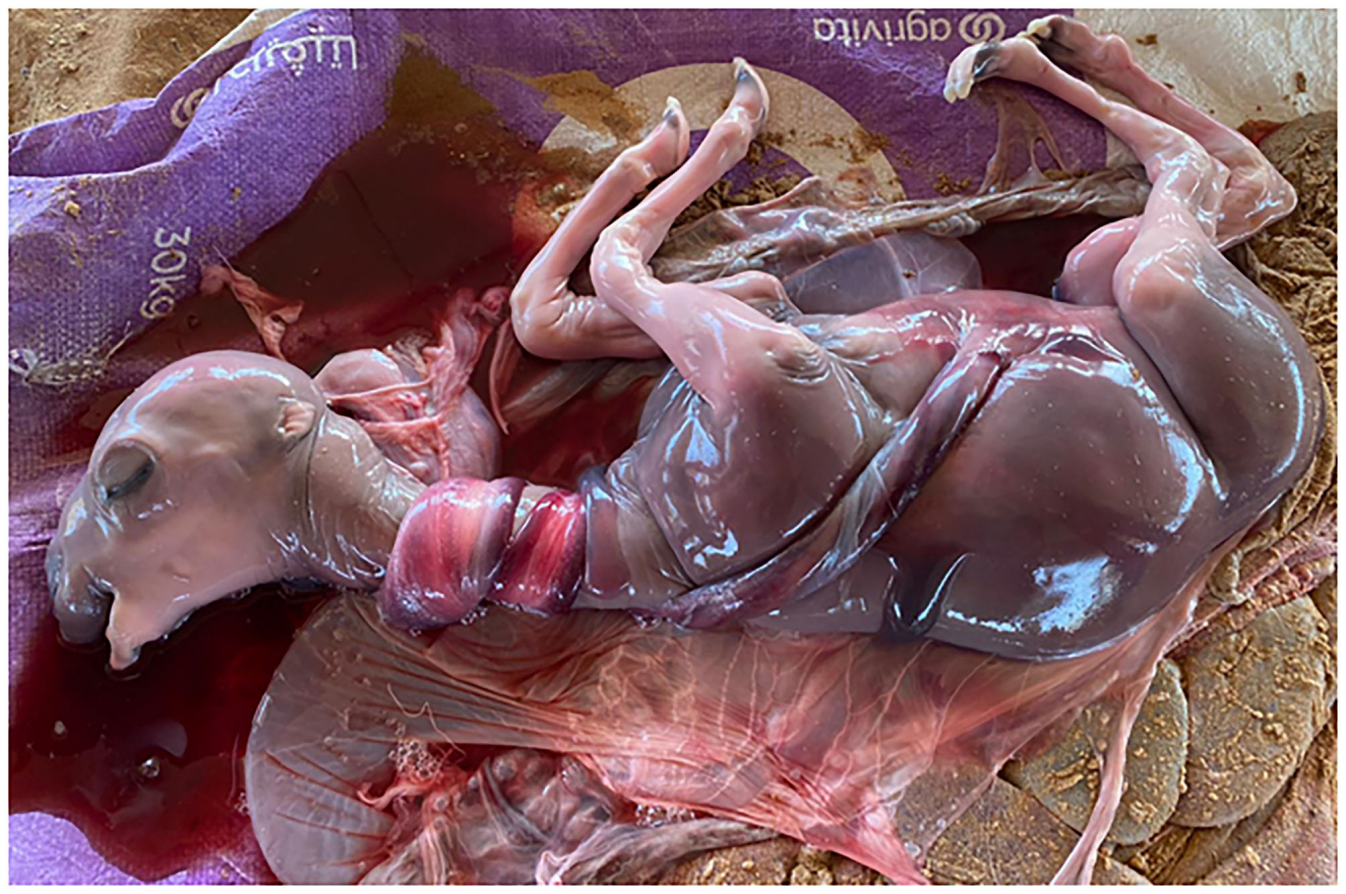
Aborted fetus at 36 weeks of gestation. Fetal death may occur because of the double-looped nuchal cord.

A fetal sac was observed on the ground in the morning. The recipient had obvious signs of abortion. On autopsy, a full-grown fetus was found. The neck of the fetus was strangulated tightly by double loops of the umbilical cord (Figure 3). There was no congenital anomaly or other malformation in the fetus. On macroscopic examination of the umbilical cord, no lesion was observed. As the recipient aborted at mid to late gestation, the brucellosis test was performed in the mother's blood and fetal tissue which was negative. A microsatellite analysis of 13 specific loci for *Camelus dromedarius* confirmed the reliability of cloned calves from donor cells (data may be provided upon request).

## Discussion

Pregnancy loss is a common ailment that affects a large number of camel pregnancies. However, there is no complete study on the incidence and causes of abortion in Camelidae. Here, we describe the first reported case of pregnancy loss in a camel by fetal death from strangulation by its umbilical cord, which is commonly known as nuchal cord. Although this is the first reported case of fetal strangulation in camel, it is likely not unique. We assume that this may be a fairly common event, although there is little supportive evidence, and to our knowledge, no direct data are reported.

The possible connotation between nuchal cord and stillbirth is unclear. Hammad et al. (10) reported that 19% of all stillbirths and 28% of stillbirths at or beyond 32 weeks were associated with umbilical cord abnormalities in humans. Most studies are in consensus that the nuchal cord is the most prevalent type of umbilical cord abnormality; however, the detrimental effects of nuchal cord on infants depend on its nature and extent, such as the number and tightness of the loops. A single or loosely wrapped nuchal cord is not associated with increased adverse perinatal outcome (11, 12). A nuchal cord that is tightly wrapped or has more than two loops may be associated with increased risk of stillbirth or compromised neonatal status at delivery (13).

The utilization of advanced reproductive biotechnologies in camels has increased substantially in recent years. Our group alone has produced hundreds of cloned pregnancies in the past 3 years (7–9), and the number of offspring produced from embryo transfer increases every year. The increased value of these pregnancies along with the accessibility of veterinary care provides the potential for routine ultrasonographic examination. In this report, we described an observation that may contribute to veterinary obstetrics and increase the understanding of late-term fetal loss. This report described one of several potential causes for the high rate of fetal loss in camels and described the first report of nuchal strangulation in the species. This observation and similar case reports should increase awareness and allow for a more detailed examination of pregnancies, with the intended result being decrease in late-term fetal loss.

In conclusion, although no nuchal strangulation reports are known in veterinary medicine, reports on human are prevalent. Ultrasonographic evaluation of pregnancy in camels at the later period of gestation is particularly difficult. No non-surgical methods exist at present to address the nuchal cord, and induction of parturition may be the only recourse. The umbilical cord may be affected by a number of abnormalities that may originate during pregnancy, labor, or delivery. It is important to investigate the underlying mechanisms of the development of cord morphology and possible pathologies associated with it, because this may provide important insights regarding fetal growth in the intrauterine environment and its impact on later life.

## Data Availability Statement

The raw data supporting the conclusions of this article will be made available by the authors, without undue reservation.

## Ethics Statement

The animal study was reviewed and approved by Management of Scientific Centers and Presidential Camels (Accession No: PC4.1.5).

## Author Contributions

Y-BS and MH designed the experiments and prepared the manuscript. XY, YJ, and PO collected the samples and reviewed the manuscript. WH supervised the project. All authors have read and approved the final version of the manuscript.

## Conflict of Interest

The authors declare that the research was conducted in the absence of any commercial or financial relationships that could be construed as a potential conflict of interest.

## Publisher's Note

All claims expressed in this article are solely those of the authors and do not necessarily represent those of their affiliated organizations, or those of the publisher, the editors and the reviewers. Any product that may be evaluated in this article, or claim that may be made by its manufacturer, is not guaranteed or endorsed by the publisher.
